# Stop Codon Usage as a Window into Genome Evolution: Mutation, Selection, Biased Gene Conversion and the TAG Paradox

**DOI:** 10.1093/gbe/evac115

**Published:** 2022-07-22

**Authors:** Alexander T Ho, Laurence D Hurst

**Affiliations:** Milner Centre for Evolution, University of Bath, Bath, United Kingdom; Milner Centre for Evolution, University of Bath, Bath, United Kingdom

**Keywords:** stop codon usage, translation termination, translational read-through, stop codon read-through, molecular evolution, genome evolution

## Abstract

Protein coding genes terminate with one of three stop codons (TAA, TGA, or TAG) that, like synonymous codons, are not employed equally. With TGA and TAG having identical nucleotide content, analysis of their differential usage provides an unusual window into the forces operating on what are ostensibly functionally identical residues. Across genomes and between isochores within the human genome, TGA usage increases with G + C content but, with a common G + C → A + T mutation bias, this cannot be explained by mutation bias-drift equilibrium. Increased usage of TGA in G + C-rich genomes or genomic regions is also unlikely to reflect selection for the optimal stop codon, as TAA appears to be universally optimal, probably because it has the lowest read-through rate. Despite TAA being favored by selection and mutation bias, as with codon usage bias G + C pressure is the prime determinant of between-species TGA usage trends. In species with strong G + C-biased gene conversion (gBGC), such as mammals and birds, the high usage and conservation of TGA is best explained by an A + T → G + C repair bias. How to explain TGA enrichment in other G + C-rich genomes is less clear. Enigmatically, across bacterial and archaeal species and between human isochores TAG usage is mostly unresponsive to G + C pressure. This unresponsiveness we dub the TAG paradox as currently no mutational, selective, or gBGC model provides a well-supported explanation. That TAG does increase with G + C usage across eukaryotes makes the usage elsewhere yet more enigmatic. We suggest resolution of the TAG paradox may provide insights into either an unknown but common selective preference (probably at the DNA/RNA level) or an unrecognized complexity to the action of gBGC.

**Significance**

Between species and within genomes, codon usage is highly variable due to a complex interplay of evolutionary forces that include mutation bias, selection, and G + C pressure. In this review, we consider the influence of each in determining the relative usage of the three stop codons (TAA, TGA, and TAG) for species across the tree of life. In doing so, we not only highlight the significant gaps in our understandings but demonstrate the utility of the stop codon exemplar for studying molecular evolution more generally.

## Introduction

There has been extensive consideration of why, within coding sequence, one codon may be used more or less than an alternative codon specifying the same amino acid, this being a cornerstone of the selectionist/neutralist debate (Knight et al. 2001). Analyses of synonymous codon usage biases have highlighted, among other things, the importance of the balance between mutation and selection and the role of translational dynamics in determining codon preferences (Andersson and Kurland 1990; Duret 2002; Chamary et al. 2006; Hershberg and Petrov 2008; Plotkin and Kudla 2011). Indeed, in many species for each amino acid, there exists an optimal codon that commonly reflects the most abundant iso-acceptor tRNA (Sharp and Li 1987; Bulmer 1991; Akashi and Schaeffer 1997; dos Reis et al. 2004). This optimal codon is also typically enriched in the more highly expressed genes.

**Fig. 1.**
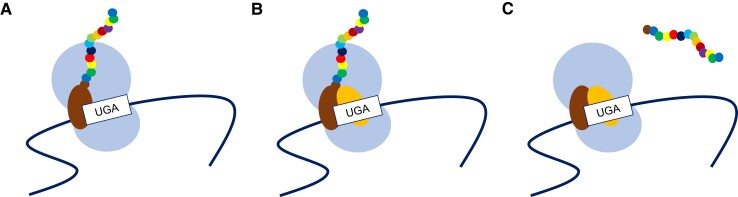
The basic mechanism of stop codon recognition by class I release factors. (*A*) The translating ribosome decodes coding sequence and recruits cognate amino-acylated tRNAs (brown) to build the growing polypeptide amino acid chain (small, colored circles). (*B*) The stop codon (UGA in this example, but typically UAA, UGA, or UAG) is recognized by, and becomes bound to, a class I release factor: RF1 or RF2 in bacterial, eRF1 in eukaryotic, or aRF1 in archaeal genomes (orange). (*C*) The binding between the release factor and stop codon begins a cascade leading to polypeptide release via the action of a class II release factor (not shown). Note that stop codons function in mRNA and hence genomic T (thymine) is replaced by U (uracil).

Most organisms also have three alternative options for the stop codon (UAA, UGA, and UAG in mRNA or TAA, TGA, and TAG in genomic sequence). Like synonymous codons, they too share the same “meaning” (Povolotskaya et al. 2012; Belinky et al. 2018). As amino-acylated tRNAs are not involved in stop codon recognition (for illustration of the process, see fig. 1), it is less obvious why selection might prefer one stop codon over another. Nonetheless, we can ask a series of questions that parallel those asked of codon usage bias. What is the role of mutation bias and neutral, or nearly neutral, evolution in determining within- and between-species variation in stop codon usage? In any given species is there an optimal stop codon and, if so, why? While the optimal sense codon for any synonymous group tends to vary between species as tRNA copy numbers vary (Duret 2002), we can also ask whether the same stop codon is optimal in all species. Many potential answers to these questions point to a role for forces that affect nucleotide content beyond the confines of stop codon usage. In this context, trends in TGA and TAG stop codon usage provide an unusual window into genome evolution as, given their identical functionality and nucleotide content, any differences in their usage requires explanation beyond a simple null model.

With direct determination of mutational profiles and extensive genome level analysis permitting analysis of both stop codon substitution rates and usage patterns, there has recently been some progress in understanding the determinants of stop codon usage. Here, we summarize recent advances in understanding the forces operating on stop codon usage emphasizing what we do now understand but also the large (and profound) gaps in understandings. We consider two sorts of comparisons. On the one hand, we have inter-species variation in usage trends where, with their extreme G + C contents, bacterial genomes are especially informative (note that G + C here and elsewhere refers to G + C content and not the GpC dinucleotide). On the other, we make use of the human genome, where intragenomic extremes of G + C content due to its isochores (relatively homogeneous domains of high/low G + C) provide evidence of similar utility. Analysis of the human genome is especially useful as we have well-resolved parameters, such as the mutational profile and recent recombination rates, along with high-quality expression data and ortholog description for closely related species. Intragenomic analysis also controls for possible mechanistic differences between taxa in stop codon recognition and release. In bacteria, for example, there are two class I release factors, RF1 and RF2, that are indispensable for stop codon recognition in all species with the standard genetic code, while in archaea and eukaryotes, there is just one (Frolova et al. 1994; Inagaki and Doolittle 2000; Jackson et al. 2012; Kobayashi et al. 2012; Rodnina 2018).

There are numerous issues related to the stop codons that we do not here investigate. For example, there exist species that do not use all three of TAA, TGA, and TAG to terminate translation, such as bacterial genomes decoded by NCBI translation table 4 (that do not use TGA) and some ciliates (e.g., *Paramecium tetraurelia* and *Stylonychia mytilis* use only TGA) (Alkalaeva and Mikhailova 2017). Why such species might not use the canonical three stop codons falls outside of our scope. It is also known that selection operates on stop codons outside of the canonical termination context. Additional in-frame stop codons (ASCs), for example, are under positive selection in some eukaryotes (but not bacteria) probably as an error-proofing mechanism to provide a second opportunity for translation to terminate should the primary stop codon be missed (Major et al. 2002; Liang et al. 2005; Adachi and Cavalcanti 2009; Korkmaz et al. 2014; Ho and Hurst 2019). Similarly, out-of-frame stop codons (OSCs) are hypothesized to be selected to mitigate the consequences of frame-shift errors should the reading frame be disrupted (Seligmann and Pollock 2004; Abrahams and Hurst 2018). We do not broach the issues of noncanonical stop codon selection in this review.

## The TAG Problem: Low Usage and Unresponsiveness to G + C Pressure

**Fig. 2.**
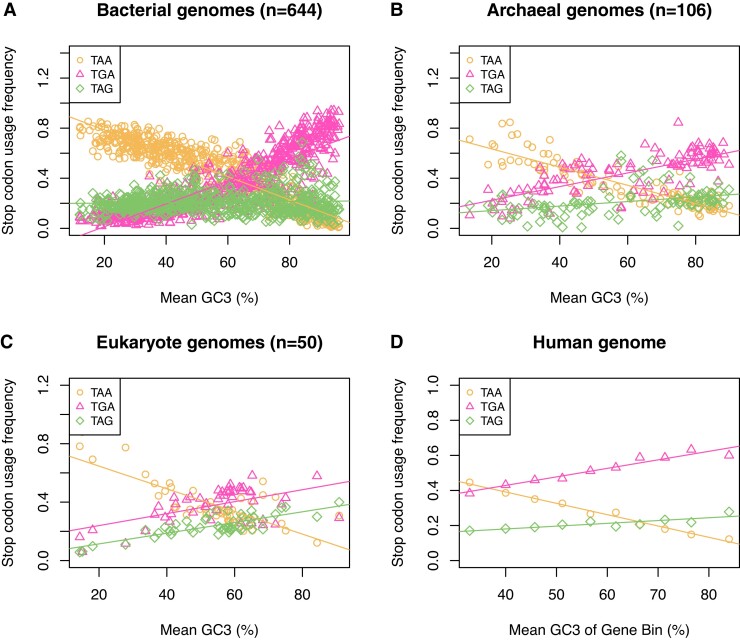
Stop codon usage (*A*) among 644 bacterial genomes, (*B*) 106 archaeal genomes, (*C*) 50 eukaryote genomes, and (*D*) among human isochores. TAA usage is negatively correlated with G + C3 content in all four analyses (Spearman’s rank; *P* < 2.2 × 10^−16^, rho = −0.92 for bacteria; *P* < 2.2 × 10^−16^, rho = −0.89 for archaea; *P* = 4.2 × 10^−7^, rho = −0.66 for eukaryotes; *P* < 2.2 × 10^−16^, rho = −0.92 within the human genome). TGA usage is positively correlated with G + C3 content in all four analyses (Spearman’s rank; *P* < 2.2 × 10^−16^, rho = 0.88 for bacteria; *P* < 2.2 × 10^−16^, rho = 0.76 for archaea; *P* = 0.0035, rho = 0.41 for eukaryotes; *P* < 2.2 × 10^−16^, rho = 0.98 within the human genome). TAG usage is uncorrelated with G + C3 content in bacteria (Spearman’s rank; *P* = 0.48, rho = −0.03). TAG usage is positively correlated with G + C3 content, but with lower absolute usage than TGA, in archaea (Spearman’s rank; *P* = 1.1 × 10^−7^, rho = 0.49), eukaryotes (Spearman’s rank; *P* = 1.1 × 10^−6^, rho = 0.64), and within the human genome (Spearman’s rank; *P* = 0.0020, rho = 0.88). Figure adapted from Ho and Hurst (2021). Species lists and underlying data can be found in the Supplementary material.

When viewed across species, codon usage has a single strong predictor (Knight et al. 2001) this being what we here call “G + C pressure” so as to not to prejudge its cause. A diagnostic of this is a correlation between G + C usage at codon third sites and some other (hopefully independent) measure of G + C content, such as G + C of introns, intergene spacer, etc. We can ask in turn whether stop codon usage is simply explained by G + C pressure. If so, explaining stop codon usage may be simple problem: whatever explains G + C pressure explains stop codon usage. If we consider the proportional usage the three stop codons in any given genome and ask how this varies between different bacteria with different G + C content, then we see that TAA and TGA behave approximately as expected: TAA usage declines with increasing G + C pressure while TGA increases (fig. 2*A*). The enigma is the behavior of TAG whose usage is both low (∼20%) and unchanging with G + C pressure, even though TGA has identical nucleotide content (Povolotskaya et al. 2012; Korkmaz et al. 2014; Ho and Hurst 2020).

The TAG problem deepens when it is noticed that across archaea and among isochores in the human genome the same three trends are seen: TAA declining, TGA increasing, and TAG either invariant or more weakly responding to G + C pressure. At first sight, archaea and bacteria look to be slightly different with TAG showing a small G + C pressure response (weak positive slope of TAG predicted by G + C pressure) in the former but not the latter. However, the bacterial data have more extreme values of G + C content and allowing for this (by comparing G + C-matched archaeal and bacterial samples), the trends seen in the two are much more similar (Ho and Hurst 2021).

**Fig. 3.**
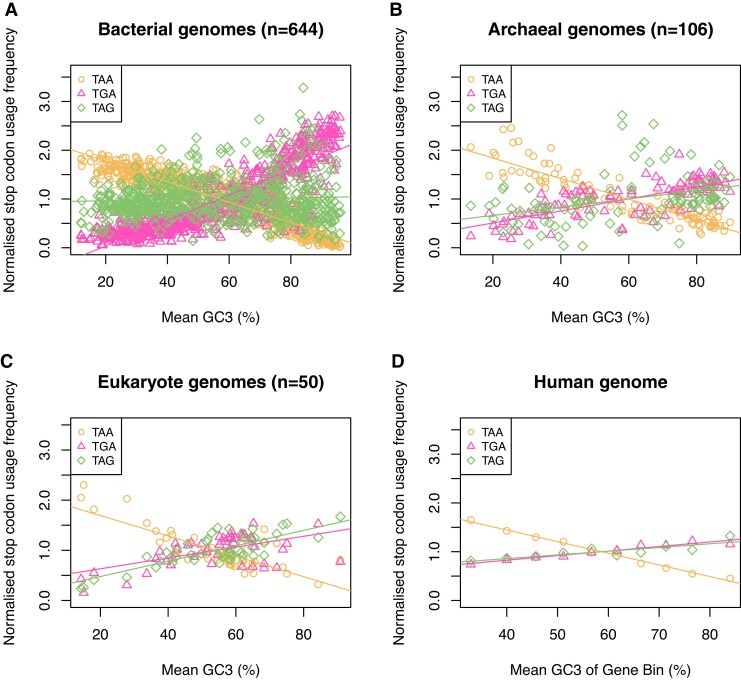
Stop codon usage normalized to the mean (*A*) between 644 bacterial genomes, (*B*) 106 archaeal genomes, (*C*) 50 eukaryote genomes, and (*D*) between human isochores. Normalization to the mean has no effect on the correlation statistics presented in fig. 2. Normalized TAA usage is negatively correlated with G + C3 content in all four analyses (Spearman’s rank; *P* < 2.2 × 10^−16^, rho =−0.92 for bacteria; *P* < 2.2 × 10^−16^, rho = −0.89 for archaea; *P* = 4.2 × 10^−7^, rho = −0.66 for eukaryotes; *P* < 2.2 × 10^−16^, rho = −0.92 within the human genome). Normalized TGA usage is positively correlated with G + C3 content in all four analyses (Spearman’s rank; *P* < 2.2 × 10^−16^, rho = 0.88 for bacteria; *P* < 2.2 × 10^−16^, rho = 0.76 for archaea; *P* = 0.0035, rho = 0.41 for eukaryotes; *P* < 2.2 × 10^−16^, rho = 0.98 within the human genome). Normalized TAG usage is uncorrelated with G + C3 content in bacteria (Spearman’s rank; *P* = 0.48, rho = −0.03). TAG usage is positively correlated with G + C3 content, but with lower absolute usage than TGA, in archaea (Spearman’s rank; *P* = 1.1 × 10^−7^, rho = 0.49), eukaryotes (Spearman’s rank; *P* = 1.1 × 10^−6^, rho = 0.64), and within the human genome (Spearman’s rank; *P* = 0.0020, rho = 0.88). Figure adapted from Ho and Hurst (2021). Species lists and underlying data can be found in the Supplementary material.

**Fig. 4.**
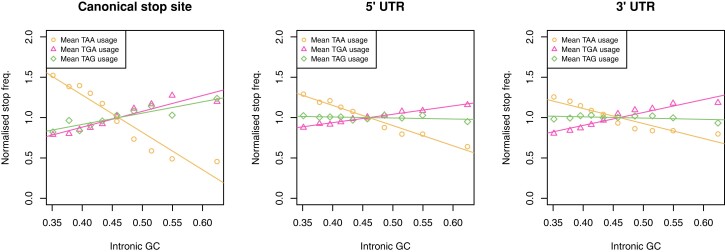
Stop codon frequencies (relative to the usage of all stops) normalized to the mean at the canonical stop site, in the 5′ UTR, and in the 3′ UTR at 10 equal-sized bins of various intronic G + C contents in the genome. Normalized TAA frequency is negatively correlated with intronic G + C content in all 3 sequences (Spearman’s rank; all *P* < 2.2 × 10^−16^, rho = −0.99 at the canonical stop site and in 5′ UTR sequences, rho = −1 in 3′ UTR sequences). TGA is positively correlated with intronic G + C content in all 3 sequences (Spearman’s rank; all *P* < 2.2 × 10^−16^, rho = 0.99 at the canonical stop site and in 5′ UTR sequences, rho = 1 in 3′ UTR sequences). TAG usage is positively correlated with intronic G + C content at the canonical stop site (Spearman’s rank; *P* = 0.0014, rho = 0.89) but is uncorrelated with intronic G + C content in both 5′ (Spearman’s rank; *P* = 0.61, rho = 0.19) and 3′ UTR sequences (Spearman’s rank; *P* = 0.10, rho = 0.55). Figure adapted from Ho and Hurst (2022). Underlying data can be found in the Supplementary material.

By considering changes in absolute stop codon usage, one assumes that there is no constraint preventing TAG usage from rising from *x*% at 0% G + C content to *x* + *y*% at 100% G + C content, just as TGA goes from *z*% at 0% G + C content to *z* + *y*% at 100% G + C content (where *z* > *x*). This in effect assumes a null in which, as GC content goes up and TAA stop codons may be switched to TGA or TAG, they are equally as likely to be switched to TGA as to TAG. An alternative null might suppose there to be something skewing this process (in favor of TGA). If so, the slopes of stop codon usage against G + C content would be affected in proportion to this skew. An alternative approach that attempts to control for this possibility is to ask about the proportional response, whereby each value is divided by the mean value for the relevant overall set of samples in question. Using this methodology, TAG and TGA usage still have very different slopes in bacteria (fig. 3) and in human 3′ and 5′ UTR sequences (fig. 4), and in both cases the distribution of TAG usage remains flat. However, in archaea and across human isochores at the focal termination site, the slopes for TGA and TAG converge.

Which method is most appropriate is contingent on what one considers to be the appropriate null model. Here, we favor the simplest (nonproportional) null model for a few reasons. First, when we look at eukaryotes TAG and TGA report two parallel lines (fig. 2*C*) suggesting that the simplest null can be valid. Second, if this is the correct null, then the proportional methodology could mislead: if the absolute usage slopes were identical but the means different, then different proportional slopes would be reported. Third, even if we cannot reject the proportional null model, this would still leave open the problem of why the mean is different, thus leaving interesting biology unresolved.

We, therefore, propose that the TAG problem may be more broadly defined as the enigmatic difference in slope between absolute TGA usage versus G + C, on the one hand, and absolute TAG usage versus G + C, on the other, the latter being much shallower. That it is seen in three independent contexts adds to the problem. That it is not replicated in analysis across eukaryotes only adds to the perplexity.

## Stop Codon Usage and Release Factor Diversity: A Genomic Red Herring?

A longstanding hypothesis to explain between-species stop codon usage in bacteria stems from the fact that bacterial translation termination at each of the three stop codons requires different molecular machinery. In bacteria, TAG is recognized uniquely by RF1 while TGA is recognized uniquely by RF2 and TAA is recognized either by RF1 or RF2 (Rodnina 2018). Early analysis observed (1) that TAA (with its broad RF-binding potential) is the most common stop codon and (2) that the TAG:TGA usage ratio positively correlated with the RF1:RF2 abundance ratio in a small sample of bacterial genomes, hence it was proposed that release factor abundance was one of the drivers driver of bacterial stop codon usage (Sharp and Bulmer 1988). Indeed, subsequent larger multi-species analyses have supported the correlations between RF1:RF2 and TAG:TGA and similarly assumed that RF abundance causes stop codon usage adjustment and not vice versa (Korkmaz et al. 2014; Wei et al. 2016). The notion that RF1:RF2 relative abundance determines stop codon usage bears obvious parallels with the idea that synonymous codon usage is determined by iso-acceptor differential tRNA abundance. As Wei et al. (2016) identified that RF2 is exceptionally low when G + C3 content is low across species, RF1:RF2 might also help explain the TAG problem. They argue that in G + C-poor regions or genomes, mutation bias favors TAA, the most G + C-poor stop codon, over both TAG and TGA. At mid-to-high G + C contents, TGA is preferred over TAG as RF2 expression levels become increasingly dominant over that of RF1.

We have since challenged this interpretation of the RF1:RF2 correlation with TAG:TGA, asking why the RF1:RF2 ratio should not instead be molded to the stop codon requirements of the genome (Ho and Hurst 2021). First, we noted that in humans and archaea there is only one release factor. That we see the same TAG problem between human isochores and across archaea (fig. 2) thus indicates that some other forces can give the TAG anomaly. Second, if RF abundance were to cause stop codon usage variation, one might predict that between-species stop codon trends in bacteria (particularly the disconnect between TGA and TAG usage) should not be repeated in noncanonical stop codon contexts where RF recognition is nonimportant. We, however, found the relative usage of TGA, TAA, and TAG in sequence immediately 3′ of genes have the same trends as seen at the canonical stop context (Ho and Hurst 2021). This is unlikely to be owing to selection for additional stop codons in the 3′ noncoding sequence for two reasons. First, while there is evidence of selection for additional 3′ in-frame stop codons (ASCs) in some single-celled eukaryotes (Ho and Hurst 2019), the same is not seen in multicellular eukaryotes or bacteria (Major et al. 2002; Korkmaz et al. 2014; Ho and Hurst 2019). Second, the same trend is also seen if we examine sequence post the first in frame stop codon (Ho and Hurst 2021). All the above points of evidence strongly suggest that we need to evoke some force other than RF diversity to explain trends in usage of TAA, TGA, and TAG.

A further corollary of the above evidence is that the better explanation for the RF1:RF2 correlation with stop codon usage is that RF abundance adapts to stop codon usage and not vice versa. This direction of the causal arrow is parsimonious for several reasons. As we outline in Ho and Hurst (2021), the molding of stop codon usage (particularly TGA<->TAG) to respond to the RF environment does not make clear evolutionary sense. As the RF hypothesis itself states that TAA is optimal due to its dual recognition by RF1 and RF2 (of which more below), there is no selective need to switch from TAA → TGA or TAA → TAG. TGA and TAG usage adjustment to match RF1:RF2 hence must theoretically proceed via net TGA<->TAG exchanges. This is significant, as, presuming that TAG → TGA and TGA → TAG cannot occur in one mutational step, any such genome-wide shift in usage must involve one step that is opposed by selection. Assuming conservation of stop codon identity then there must be TAA → TGA or TAA → TAG either of which is deleterious. Under the RF hypothesis, then, it is unclear why selection should favor net genome-wide TGA<->TAG shifts in any scenario.

With the RF hypothesis seemingly unparsimonious to explain species differences among bacteria, and irrelevant when we consider eukaryotes and archaea (which possess just one RF), arguments for stop codon usage trends being driven by RF diversity appear to be a red herring (i.e., a distraction from the main explanation). For the rest of this review, we consider the myriad of factors that likely act to shape the stop codon usage of all species. We consider the roles of mutation bias, selection, and biased gene conversion, discussing how these too might vary between species.

## Null Mutational Models Cannot Alone Explain Within- or Between-Genome Variation in Stop Codon Usage

For stop codon usage, as with synonymous codon usage bias, the simplest null would be one of neutral evolution coupled to mutation bias. Originally, the variation in G + C content between species was indeed assumed to reflect the mutational process, assuming G + C content at third site to be approximately neutral and reflective of mutational biases (Muto and Osawa 1987; Knight et al. 2001). However, now that we can measure mutational biases directly the assumption that G + C-rich genomes and genomic regions (such as the G + C-rich isochores in humans) are solely a consequence of mutational bias alone is no longer defendable.

From analysis of mutations seen in parent offspring sequencing, mutation accumulation (MA) lines or rare SNPs, across both eukaryotes and prokaryotes mutation bias appears to be very commonly G + C → A + T biased (Smith and Eyre-Walker 2001; Lynch et al. 2008; Hershberg and Petrov 2010; Hildebrand et al. 2010; Long et al. 2018). Importantly, this commonality applies just as much to G + C-rich genomes as to G + C-poor ones (Hershberg and Petrov 2010; Hildebrand et al. 2010). Hence, G + C-rich genomes sit far away from their A + T-biased mutational equilibrium (Long et al. 2018). Stop codon usage in part reflects this deviation from mutational null. We would expect under a simple mutation bias null for TGA and TAG to be universally and equally rare and TAA to be the most abundant. However, this null mutational model fails to explain either within- or between-genome variation. Notably TAA usage, while indeed high in G + C-poor bacterial genomes, is low in G + C-rich ones (fig. 2) despite the profile of mutation bias being commonly G + C → A + T biased (Hershberg and Petrov 2010; Hildebrand et al. 2010).

**Fig. 5.**
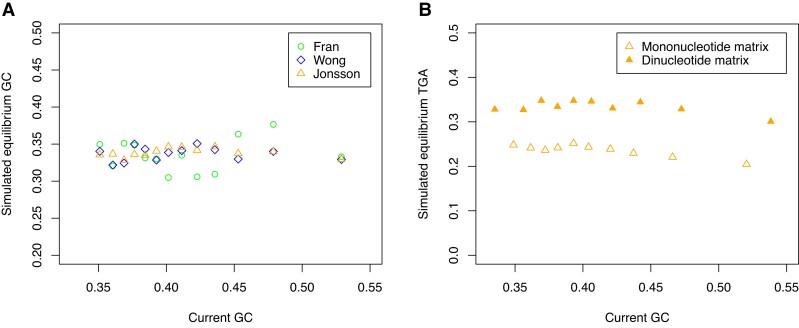
Simulated equilibrium (*A*) G + C content and (*B*) TGA usage plotted against the current G + C content of the windows from which the mutation spectrum was estimated. Panel A is reproduced with permission from Smith et al. (2018) (the original figure is available open access at: https://doi.org/10.1371/journal.pgen.1007254.g004) and shows equilibrium G + C estimates from three sources of human de novo mutations. Panel B is reproduced from Ho and Hurst (2022) and illustrates equilibrium TGA usage (relative to TAA and TAG usage) estimated from the Jonsson et al. (2017) dataset of human de novo mutations. This is done either employing a 4 × 4 mononucleotide mutational matrix or from a 16 × 16 dinucleotide matrix with Markov process to define *k*-mer equilibrium content.

Perhaps the best current data come from humans as here, by parent offspring sequencing, we have an exceptional view of the mutational process. We can then, for example, ask whether in G + C-rich isochores (with an abundance of TGA) the mutational profile is more A + T → G + C biased than in the G + C-poor isochores. Strikingly, a G + C → A + T mutation bias is approximately invariant to isochore G + C content (Smith et al. 2018; Ho and Hurst 2022; fig. 5), but nonetheless TGA usage increases with local G + C with TAA decreases (as seen in fig. 2). In this case, more complex mutational biases (e.g., high rates of CpG → TpG (Duncan and Miller 1980; Sved and Bird 1990; Roberts and Gordenin 2014) which could generate new TGA stop codons) also cannot account for the decline in TAA usage and increase in TGA usage as local G + C content increases (Ho and Hurst 2022).

**Fig. 6.**
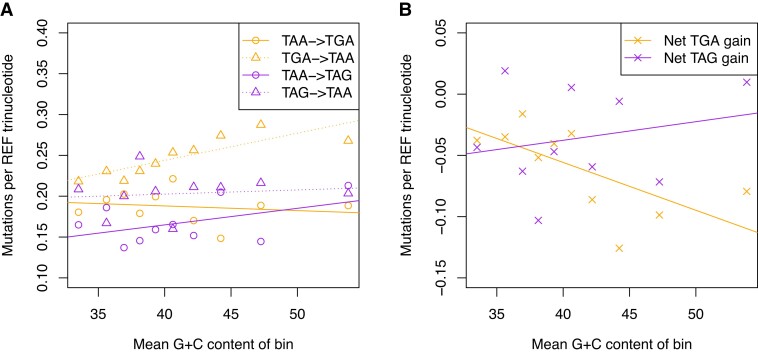
Rates of point mutations leading to (*A*) TAA<->TGA and (*B*) TAA<->TAG trinucleotide changes derived from the Jonsson et al. (2017) dataset (*n* = 108,778). De novo mutations were partitioned according to their surrounding (10 kb) G + C content into 10 equal bins. Mutations causing TAA->TGA (Spearman’s rank; *P* = 0.51, rho = −0.24, *n* = 10) and TAA->TAG (Spearman’s rank; *P* = 0.51, rho = 0.24, *n* = 10) changes are invariant to G + C pressure. Net TGA and net TAG refer to the TAA->TGA rate minus the TGA->TAA rate and the TAA->TAG rate minus the TAG->TAA rate, respectively. Net TAG gain is invariant to G + C pressure (Spearman’s rank; *P* = 0.97, rho = 0.018, *n* = 10). Net TGA gain is negatively correlated with G + C pressure (Spearman’s rank; *P* = 0.028, rho = −0.71, *n* = 10). Underlying data can be found in the Supplementary material.

To assess the extent to which mutation bias might explain the TAG problem we may refine our analysis to consider only point mutations that lead to TAA<->TGA and TAA<->TAG trinucleotide changes. Indeed, whether TGA or TAG will be more frequent than the other could depend on whether TAA will mutate equally or unequally to TGA or TAG. However, in contrast to the possibility that mutations from TAA to TGA or TAG cause the TAG problem, we find mutations causing TAA->TGA (Spearman’s rank; *P* = 0.51, rho = −0.24, *n* = 10) and TAA->TAG (Spearman’s rank; *P* = 0.51, rho = 0.24, *n* = 10) changes to be approximately invariant to G + C pressure (fig. 6*A*). Perhaps the more complete test is to consider net TGA gain and net TAG gain, these metrics being the TAA->TGA rate minus the TGA->TAA rate and the TAA->TAG rate minus the TAG->TAA rate, respectively. That we find net TAG gain to be invariant to G + C pressure (Spearman’s rank; *P* = 0.97, rho = 0.018, *n* = 10) and net TGA gain negatively associated with G + C pressure (Spearman’s rank; *P* = 0.028, rho = -0.71, *n* = 10) (fig. 6*B*) agrees with all the above results that mutation bias cannot explain stop codon usage trends.

Analysis of the human mutational profile also indicates that trinucleotide frequencies are closer to mutational equilibrium in G + C-poor isochores than G + C-rich ones (Ho and Hurst 2022). At G + C-poor isochores, we can compare equilibrium estimates of TAA, TGA, and TAG trinucleotides to their relative usage at the canonical stop site to assess how well the mutational profile predicts what is seen in termination contexts. Deviation at the stop site from the predicted relative frequencies of TAA, TGA, and TAG trinucleotides would indicate the presence of nonmutational forces influencing stop codon usage. Using the same human dinucleotide mutational matrix as in Ho and Hurst (2022), we estimate the equilibrium relative usage of TAA to be 43.0%, TGA to be 32.5%, and TAG to be 24.5% in the bottom 20% of human genes by G + C content. Despite their shared nucleotide content, mutational preferences at the dinucleotide level appears to somewhat discriminate between TGA and TAG, perhaps because CpG → TpG mutations are very common (Duncan and Miller 1980; Sved and Bird 1990; Roberts and Gordenin 2014), and hence could begin to explain the absolute differences in their usage. However, in the same set of G + C-poor genes, the stop codon usage at the canonical stop site is 38.4% TAA, 42.0% TGA, and 19.5% TAG. This in turn suggests that in G + C-poor domains usages are reasonably close to, but distinct from, mutational expectations.

While in G + C-poor regions complex mutation bias takes us some way to understanding the lower usage of TAG, mutation bias fails to explain the differing response of TAG and TGA to G + C pressure as mutation bias does not covary with G + C content. As TGA usage at mutational equilibrium in a trinucleotide model (e.g., allowing for CpG mutability) is invariant as a function of isochore G + C (Ho and Hurst 2022), a mutational model evoking loss of the focal stop and gain of a downstream one in 3′ UTR, can thus also not explain the trends. Consequently, the usage of all three stops is far from mutational equilibrium in G + C-rich isochores. Using the same dataset, we estimate the equilibrium relative usage of TAA to be 42.6% (compared to an observed usage of 13.6%), TGA to be 32.0% (compared to 63.5%), and TAG to be 25.4% (compared to 22.9%) in the top 20% of human genes by G + C content. Coupled with evidence for near-universality of a G + C → A + T mutation bias (Smith and Eyre-Walker 2001; Lynch et al. 2008; Hershberg and Petrov 2010; Hildebrand et al. 2010; Long et al. 2018), mutation bias provides no robust explanation of the TAG problem and only partially explains why organisms differ in the G + C content more generally.

## The Three Stop Codons Are Not Selectively Equivalent

### Genomic Evidence Supports TAA Optimality

Given that a mutational neutral null appears to be insufficient in explaining within- or between-genome variation in stop codon usage, as with synonymous codon usage bias one might suspect that selection has some role in stop codon usage. Several approaches have been taken to determine which stop codon might be optimal. To a first approximation, they all concur that TAA is universally optimal.

The first method considers differential usage in highly expressed genes versus lowly expressed genes. This assumes that the costs of translational error are higher in highly expressed genes (see trends in synonymous codon usage). Across bacteria and in the human genome, TAA is relatively enriched in highly expressed genes suggesting a selective advantage (Korkmaz et al. 2014; Trotta 2016; Ho and Hurst 2020). In yeast, the same is observed (Trotta 2013).

The second method considers enrichment allowing for biases in the usage of dinucleotides within any given genome (note this is observed usage not the mutational profile). Against dinucleotide-controlled null models, it is TAA (and not TGA nor TAG) that is most enriched across bacteria, eukaryotes, and archaea (Ho and Hurst 2021). The third method considers trends in enrichment compared to nucleotide null as a function of effective population size (N_e_), assuming that when N_e_ is high selection is more efficient and thus enables organisms to be closer to a selectively optimal state (Ohta 1992; Lynch 2007). Such methods come with all the necessary caveats that N_e_ is hard to estimate (but with mutation rate and polymorphism data, it is now possible). To date, this has been done across eukaryotes in a phylogenetically controlled manner with TAA enrichment correlating positively with N_e_ (Ho and Hurst 2020).

A final method considers trends in stop codon substitution (i.e., fixation events) using species trios. Such a method can detect differences in relative substitution rates (e.g., TGA → TAA per TGA vs. TAA → TGA per TAA) and so infer the extent to which each is conserved (Belinky et al. 2018). Note that this is not the same as the mutational analysis as that considers just the rates of origination not the rates of origination and fixation. This method finds a bias to TAA conservation near universally (Belinky et al. 2018). However, a problem we return to below, is that this method, also reports TGA conservation in mammals (Belinky et al. 2018).

Almost all methods hence concur on universal TAA optimality. Why then might stop codons have different fitness consequences and is there evidence that any such effects mediate within- or between-genome variation? The dominant models for TAA optimality all point to its resilience in the face of errors as the probable cause, the errors in question being either mutational, mistranscriptional, or owing to misreading/misprocessing.

### TAA Is More Robust to Mutation and Mistranscription Events than TGA and TAG

Perhaps the most immediately noticeable difference between the three stop codons is the differing nucleotide compositions of TAA, TGA, and TAG. This is significant as any selective force that molds stop codon usage must commonly proceed via stop codon switch events, that is, TAA<->TGA, TAA<->TAG, and TGA<->TAG, as sense codon intermediate states at the canonical termination site are unlikely to be tolerated. TAA is unique in being robust to two mistranscription (or mutational) events, that is, TAA → TGA or TAA → TAG (Ho and Hurst 2020). TGA → TAA and TAG → TAA switches are similarly resilient to mistranscription or mutation, but no single nucleotide change permits TGA<->TAG (we assume that double mutants are extremely rare). Not only might TAA be optimal for this reason, but TGA<->TAG switches must proceed via TAA regardless of whether TGA or TAG is optimal. Mutation is probably too rare a process to select for TAA via mutational robustness, however whether much more common mistranscription events could select for TAA is unresolved—see, for example, the rates in *Escherichia coli* (Lee et al. 2012; Traverse and Ochman 2016; Meer et al. 2020) and *Caenorhabditis elegans* (Denver et al. 2004, 2009; Gout et al. 2013; Meer et al. 2020).

### TAA Is the Least, While TGA Is the Most, Prone to Molecular Errors

**Fig. 7.**
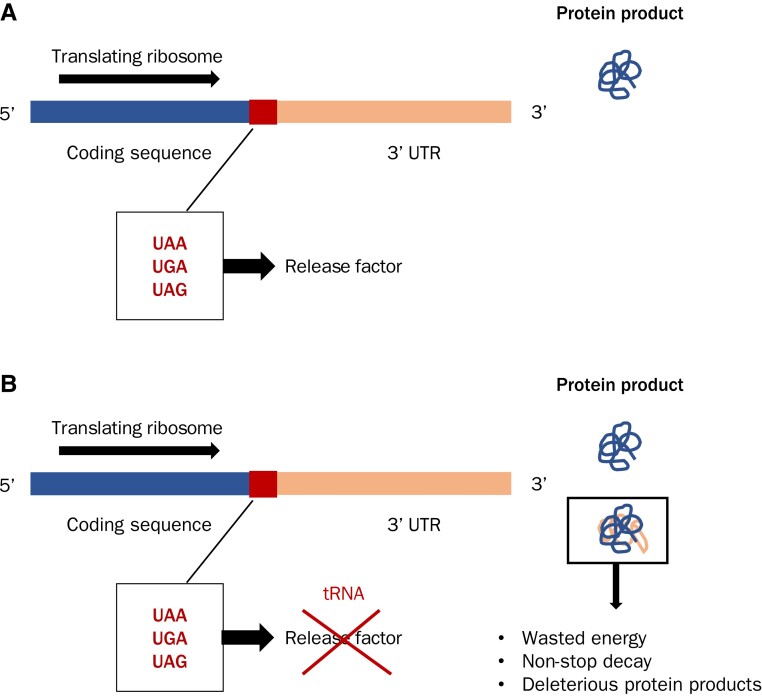
The mechanistic basis of translational read-through. (*A*) Canonical termination occurs when the stop codon is recognized by its cognate release factor. Only coding sequence is translated to build the polypeptide amino acid chain. (*B*) Translational read-through occurs when the stop codon is missed by the termination machinery, often due to the erroneous binding of a near-cognate tRNA to the stop codon (Roy et al. 2015; Beznoskova et al. 2016). This results in the translation of 3′ UTR sequence until the next in-frame stop codon or until the ribosome reaches the polyA+ tail, triggering nonstop decay.

The selective hypothesis that has garnered the most attention is that stops codons differ in their susceptibility to mistakes during gene expression. With stop codons, the most associated such molecular error is the failure to terminate translation, known as either translational read-through (TR) or stop codon read-through (SCR). Here, we will refer to this phenomenon as TR. When TR occurs the stop codon is missed by the translational machinery, typically due to erroneous misreading of the stop codon by a near-cognate tRNA, leading to unintended translation of the 3′ UTR that continues until the next in-frame stop codon or the polyadenylation signal (fig. 7).

TR is most often deleterious for several reasons. At the very least, C-terminal extension is an unnecessary energetic waste (Wagner 2005) and, in more severe cases, might lead to problems with localization and export (Falini et al. 2005; Hollingsworth and Gross 2013), aggregation (Vidal et al. 1999, 2000), or stability (Clegg et al. 1971; Namy et al. 2002; Pang et al. 2002) of the final protein product. Should translation reach the polyA+ tail, TR can also trigger degradation of both mRNA and protein (Dimitrova et al. 2009; Klauer and van Hoof 2012). To mitigate these consequences, we expect selection to reduce TR rates. In order of decreasing TR susceptibility, the order appears to be TGA ≫ TAG > TAA across bacterial (Roth 1970; Strigini and Brickman 1973; Ryden and Isaksson 1984; Parker 1989; Meng et al. 1995; Sanchez et al. 1998) and eukaryotic (Geller and Rich 1980; Parker 1989; Cridge et al. 2018) species. Stop codon switches that lower the TR error rate (TGA → TAG, TGA → TAA, TAG → TAA) could hence be favored by selection across taxa.

That TAA is the least error-prone stop codon variant makes it a strong candidate for optimality. But can we be confident that TR rather than possible other advantages of TAA (such as mistranscriptional robustness) is the core to its selective optimality? A strong clue comes from nucleotide preferences immediately proximal to the stop codon. The sequence involved in modulating TR rate likely extends for at least 6 nucleotides downstream of the stop codon for fine tuning of ribosomal interactions (Bossi and Roth 1980; Namy et al. 2001; Wei and Xia 2017; Cridge et al. 2018) and here we find TR-associated nucleotide combinations to be rare in highly expressed genes (Ho and Hurst 2020). It is the +4 nucleotide, however, that is most influential. It is important, therefore, that genes terminating with the most TR-prone context TGAC (Cridge et al. 2018) are underrepresented in eukaryotic genomes (Cridge et al. 2006). A second clue comes from sequence conservation. If TR truly does generally result from error, there is no reason why the sequence downstream of the stop codon (and before the first in-frame ASC) should be conserved. In contrast, if the read-through is functional, then we expect a lower rate of sequence evolution between the primary stop codon and the ASC than 3′ of the ASC. Li and Zhang (2019) tested this hypothesis in Drosophila and yeast by defining and comparing two regions: region 1 being the sequence between the canonical stop and first ASC, and region 2 being the sequence between the first ASC and second ASC that should be untranslated except for rare events of double TR. In both organisms, they find no evidence to support region 1 sequences being more conserved than region 2 sequences (Li and Zhang 2019).

For the above reasons, TAA is thought to be the optimal stop codon for all species for its low relative TR rate. However, one piece of evidence is, in this context, unresolved. In yeast, there exist selectively preferred ASCs in 3′ UTR, enriched at codon site +3 downstream of the canonical termination codon (Liang et al. 2005). This suggests that read-through happens and selects for a second stop codon. Curiously the conserved second stop codons are enriched for genes terminating TAA (Liang et al. 2005). *A priori*, TAA is expected to have the lowest read-through rates and hence not expected to be associated with conserved ASCs. One possible explanation is that these extra stop codons reflect increased read-through following prion upregulation that forces read-through (Wickner et al. 1995; Liebman and Chernoff 2012). If such read-through were particular to TAA, then the circle could be squared. We note too that, while TAA optimality seems universal the mechanistic underpinning of this is not at all clear. As described above, in bacteria, this was ascribed to TAA binding both RF1 and RF2 (Sharp and Bulmer 1988), but the universality suggests that this is an unnecessary model.

Just because TAA is generally optimal it does not follow that selection need favor TAA in all cases. There can be occasions when read-through might be employed as part of a sophisticated mechanism that is favorable, not deleterious. The C-terminal extension of polypeptides by TR, for example, could theoretically add new signals and domains to proteins to be viewed by natural selection (Dunn et al. 2013; Schueren and Thoms 2016). Situations such as these are known as functional read-through (FTR) and are described across the tree of life (see Schueren and Thoms (2016) for a thorough review). Perhaps the best example comes from viral genomes that use FTR to improve the coding capacity of their very small genomes (Firth and Brierley 2012). In tobacco mosaic virus, for example, TR of the TAG stop codon of the RNA replicase transcript allows the virus to yield two isoforms from one gene (Pelham 1978). In humans, a well-described example of FTR allows a 22 amino acid extension to vascular endothelial growth factor A (VEGFA) to reverse its function from proangiogenic to antiangiogenic (Eswarappa et al. 2014). The best studied metazoan with regards to FTR is the fruit fly, where ribosomal profiling has estimated ∼300–350 candidates in *Drosophila melanogaster* of which 8 were experimentally confirmed (Dunn et al. 2013). The C-terminal extensions in these cases included transmembrane domains, nuclear localization signals, a PTS1, and a prenylation signal (Dunn et al. 2013).

If TR were to be commonly functional, however, one might expect TGA stop codons (the universally leakiest stop) to be selectively preferred. To date, there is no evidence to suggest FTR is particularly common in complex organisms, perhaps because FTR is rather unnecessary in larger genomes which are not so constrained in their coding capacity (Schueren and Thoms 2016). Indeed, in silico analysis of the stop codon context of 200,000 human transcripts returned only 57 TR candidates (Schueren et al. 2014). This is not what is expected were TR to be regularly beneficial. Even in Drosophila, with its ∼300 candidate transcripts for FTR (Dunn et al. 2013), such numbers are orders of magnitude below what is needed to support genome-wide selection for stop codons that promote TR.

There is a second, if speculative, model that proposes that TR is beneficial for reasons beyond extending protein ends. This states that low-level, but consistent, TR is required for gene regulation and mRNA quality control by controlling ribosomal queuing (Yordanova et al. 2018). Under this model, translating ribosomes read past the stop site and eventually stall, translation being inhibited when the ribosome queue backs up to the stop codon (Yordanova et al. 2018). The rate of TR coupled with the length of sequence to the ribosomal stall site hence might define the number of times the mRNA can be translated. There is evidence for this at the AMD1 locus in humans (Yordanova et al. 2018), however it remains unknown how widespread a mechanism like this might be. If it is common, it could theoretically affect stop codon usage due to their different TR rates, and hence different ribosomal queuing rates, which could lead to the fine tuning of TAA, TGA, and TAG frequencies. It is for this reason that Seoighe et al. (2020) consider this model as a potential explanation for the apparent conservation of TGA stop codons in mammals. We return to the issue of mammalian exceptionalism later.

## G + C-Biased Gene Conversion Acts Antagonistically to Selection and Mutation Bias to Promote TGA Usage

Perhaps the most striking conclusion of the above is that, while we can discern TAA optimality, the effects of TAA optimality appear modest: despite TAA optimality its usage at the canonical termination position declines with G + C pressure and TAA optimality appears to have little relevance to the TAG problem. Similarly, the enrichment of TAA in highly expressed genes is modest compared with G + C pressure. We can detect TAA enrichment across species as a function of N_e_ but again the effect is quite modest (Ho and Hurst 2020). Perhaps this is most in evidence when comparing TAA abundance at the focal termination site across species/isochores to TAA usage in locations when it cannot be employed as a stop codon, the trends being almost identical in the two classes (Ho and Hurst 2019, 2021). This all suggests that TAA optimality is a sideshow (or the icing on a cake) to full understanding of trends in stop codon usage. It also questions what other forces might be operating that could explain the trends in TAA, TGA, and TAG usage.

If TAA truly is universally optimal, then there might be lessons to be learned in apparently contradictory examples. In mammals, TGA stop codons are not only high in frequency but appear to be highly conserved, even more so than TAG and, surprisingly, TAA (Belinky et al. 2018; Seoighe et al. 2020). Interrogation of stop codon usage and substitution rates has revealed this phenomenon may be primarily driven by highly compartmentalized TAA to TGA bias in domains of high G + C content (Ho and Hurst 2022; fig. 2). This is particularly interesting given the spatially structured “isochore” nature of base composition in mammalian genomes (Bernardi 1993; Eyre-Walker and Hurst 2001). The current best explanation for isochore structure is G + C-biased gene conversion (gBGC), a process through which mismatches during heteroduplex formation in meiotic recombination are resolved in a G + C-favored manner (Galtier et al. 2001; Duret and Galtier 2009). As gBGC is tightly coupled to recombination, G + C-rich alleles receive the greatest fixation advantage in highly recombining sequences, possibly even when deleterious (Galtier et al. 2009).

Could mammalian TGA (a G + C-rich stop codon) usage and compartmentalized TAA → TGA substitution bias be explained by gBGC? Several pieces of evidence are supportive such as the observation that autosomal size, which correlates negatively with recombination rate and G + C content, predicts high TGA usage in smaller, more recombinogenic chromosomes (Ho and Hurst 2022). TAA → TGA substitution rate also correlates positively with local recombination rate assayed from parent–offspring trios (Ho and Hurst 2022). Covariance between TAA → TGA substitution rate and G + C content also appears most evident in isochore-structured genomes (including birds) consistent with the possibility that they share the same underlying forces (Ho and Hurst 2022). Indeed, birds and mammals are unique in being known to have a strong A + T → G + C conversion bias that accords with the gBGC model. In humans, for example, ∼70% of G + C:A + T mismatches are resolved in favor of the G + C residue (Halldorsson et al. 2016). The gBGC model has no problem explaining why stop codon trends are seen both at the focal stop and in noncoding sequences as it does not depend on termination functionality.

Given that selective and mutational hypotheses for TGA conservation are unparsimonious, for mammals at least it hence appears that gBGC offers the best explanation for TGA conservation and its focus in high G + C isochores. We note that this is an unusual case history as TGA is unfavored by the mutation bias (G + C → A + T) and selection (most probably for TAA and reduced TR). Consequently, there is only one currently known force that can explain TGA enrichment at the focal stop codon in G + C-rich domains, this being gBGC. As TGA possesses a higher intrinsic TR error rate than TAA (Cridge et al. 2018), gBGC appears also to be fixing deleterious mutations.

While gBGC could potentially explain TGA conservation in mammals, what to expect from the gBGC outside of this example is unclear. Is gBGC universal throughout the tree of life? In yeast, the best evidence from tetrad sequencing suggests a very weak bias at best possibly even in the opposite direction (Mancera et al. 2008; Duret and Galtier 2009; Liu et al. 2018, 2019). While in humans and birds, the bias, per event, is ∼60–70%, in yeast the current best estimate is a bias of 50.03, not significantly different from the null of 50% (Liu et al. 2017). What about bacteria? Could gBGC explain trends seen between-genomes as well as within? Whether gBGC operates in bacteria remains an open issue (Lassalle et al. 2015), and further work investigating complex gBGC preferences in these groups is needed. Arguing against gBGC is the finding that G + C-rich bacterial genomes reside above mutation equilibrium even if not recombining (Hildebrand et al. 2010). The observation of some correspondence between a nonhomologous end joining double strand repair pathway and higher G + C content is worthy of further scrutiny (Weissman et al. 2019).

## Unraveling the TAG Problem: A Window into Complex *k*-mer Trends?

The sequential consideration of mutation bias, selection, and G + C pressure in determining stop codon usage primarily focuses on TAA and TGA stop codons. The omission of TAG reflects its nontypical behavior in response to G + C pressure. Any mutational or simple fixation bias (be it gBGC or selection for higher G + C content) predicts that trends in TGA and TAG stop codon usage should be the same due to their identical nucleotide content. Across bacterial and archaeal taxa and across isochores within genomes this is not seen, TGA reliably correlates positively with G + C content while TAG is underused and unresponsive to G + C pressure (fig. 2; Korkmaz et al. 2014; Trotta 2016; Ho and Hurst 2019): How may we attempt to resolve this? In addition, why is the trend across eukaryotes different?

From a mutational perspective, we may utilize data from human family trios for scrutiny of more complex mutational profiles (as above). Analysis of a mutational matrix of such de novo mutations facilitates the calculation of mutational equilibrium frequencies for any given nucleotide, dinucleotide, or trinucleotide which can then be compared against fixed frequencies to elucidate deviations from mutational null. Equilibrium TAG content in humans is indeed lower than TGA content, suggesting that a more complex mutational bias at least partially explains its low usage (Ho and Hurst 2022). Strikingly, however, TAG usage in G + C-poor isochores closely resembles equilibrium whereas this is not true in G + C-rich domains (Ho and Hurst 2022). Some kind of fixation bias needs to be evoked. As TAG and TGA have the same mononucleotide content we seem to be left having to evoke, nonmononucleotide (e.g., dinucleotide or trinucleotide or larger) level selection or an added layer of complexity to gBGC that goes beyond a simple A + T → G + C conversion bias.

In our recent study, we investigated the nature of the fixation bias by assigning a fixation bias “boost” score to each trinucleotide based upon the difference between its observed frequency and the predicted mutational equilibrium (derived from a dinucleotide mutational matrix) in G + C-rich domains (Ho and Hurst 2022). We found that TGA consistently receives a higher fixation boost than TAG. Indeed, trinucleotides may be grouped by their G + C content such that completely G + C-poor trinucleotides such as AAA may be assigned to the 0% G + C group, AGA may be assigned to the 33% group, etc. We found that the order of trinucleotides by “G + C boost” is highly consistent within each G + C class (0%, 33%, 66%, and 100%) across different classes of noncoding sequence (Ho and Hurst 2022). Notably, within the 33% G + C content class (trinucleotides with two As or Ts and 1 G or C), fixed TGA frequencies are seen far above its mutational equilibrium in 3′ UTR, 5′ UTR, introns, enhancers, etc., while TAG and TAC are always less affected by whatever fixation bias is at play (Ho and Hurst 2022). These results support the possibility of a consistent trend for a fixation bias, at least in humans, that can only be evidenced at higher resolution than mononucleotide level.

What might cause such a complex fixation bias? One possibility is some even more complex set of context dependencies of mutational biases not so far considered. However, our dinucleotide model of expected frequencies in domains of low G + C very accurately predicts observed frequencies from mutation bias alone (Ho and Hurst 2022), so this seems unlikely. As regards selection, many possibilities are imaginable but to date none seem particularly compelling. There may, for example, exist selection against TAG’s component dinucleotides (Ho and Hurst 2022). TA, for example, might be avoided to avoid transcription initiation sites (TATA in eukaryotes and “Pribnow” boxes in prokaryotes). Where this to be important, however, one might expect to see similar selection against, and low abundance of, TAA stop codons too. Other ideas have included more general DNA structural hypotheses such as TA being avoided to protect chromatin structure as A + T-rich DNA is concentrated in nucleosome free regions (Burge et al. 1992). This, however, cannot explain the stop codon usage trends being the same in bacteria as seen across the human genome as the former do not possess nucleosomes but do avoid TAG. Indeed, while any selective hypothesis must fit many different species (prokaryotic and eukaryotic), it must also involve selective coefficients that are strong enough to explain all trends.

For gBGC to explain the TAG, enigma requires amendments to the current assumptions. One possibility is that gBGC is better at recognizing mismatches at certain residues than others or that the form of the bias is dependent on the *k*-mer context. In the former model, an unrecognized mismatch is resolved in mitosis but with no bias. In the second model, all meiotic mismatches are recognized but the bias differs. There is some evidence for the latter (Halldorsson et al. 2016). Either way, it is possible that net TNA conversion bias would be different from that for TAN. If so gBGC could potentially fix more TAA to TGA mutations than TAA to TAG, for example. While promising, for complex gBGC to explain stop codon trends across taxa more generally this order of trinucleotides would have to be consistent between all organisms showing the TAG problem. Scrutiny of the across eukaryote trends (and the potential lack of TAG problem) may be a means to progress as gBGC seems to be variable in effect across eukaryotes. Examination of the context of gBGC events through tetrad sequencing or sperm typing is a high priority.

All things considered there appears to be something profound about genome evolution that we do not currently understand. From analysis of the low TAG usage at the canonical stop we have identified the TAG problem, a more general low usage and one nonresponsive to G + C pressure in many comparisons (bacteria, archaea, human, at the focal stop, and elsewhere). The mystery is compounded by the observation that the trend across eukaryotes appears to be different. As we do not have a coherent explanation, we suggest that this be considered the TAG paradox. Unraveling the TAG paradox, given its appearance throughout the tree of life, may well provide a window into a previously unrecognized world of unexplained trends in *k*-mer usage that, we suggest, must throw light onto currently not well-understood forces behind stop codon usage and genome evolution more generally.

## Supplementary Material

evac115_Supplementary_Data

## Data Availability

All relevant data may be found within the cited literature. Species lists and data underlying all the presented figures are provided in the supplemental information.
